# Consecutive Ink Writing of Conducting Polymer and Graphene Composite Electrodes for Foldable Electronics-Related Applications

**DOI:** 10.3390/polym14235294

**Published:** 2022-12-03

**Authors:** Heechan Lee, Youngdo Kim, Jiwoo Kim, Su Young Moon, Jea Uk Lee

**Affiliations:** 1 Department of Advanced Materials Engineering for Information and Electronics, Integrated Education Institute for Frontier Science & Technology (BK21 Four), Kyung Hee University, 1732 Deogyeong-daero, Giheung-gu, Yongin-si 17104, Republic of Korea; 2 Samsung Display Co., Ltd., #1 Samsung-ro, Giheung-gu, Yongin-si 17113, Republic of Korea; 3 Chemical & Process Technology Division C1 Gas & Carbon Convergent Research Center, Korea Research Institute of Chemical Technology (KRICT), 141 Gajeongro, Yuseong, Daejon 34114, Republic of Korea

**Keywords:** conducting polymer, graphene, composite, electrode, 3D printing

## Abstract

For foldable electronic devices of the future, most components should have very good flexibility and reliability to maintain electrical properties even under repeated deformation. In this study, two types of inks for conducting polymer and graphene were simultaneously printed on flexible plastic substrates via the newly developed consecutive ink writing (CIW) process for the formation of composite electrodes of foldable electronic devices. To consecutively print conducting polymer ink and graphene ink, a conventional three-dimensional (3D) printer was modified by installing two needles in the printer head, and the two inks were printed through the nozzle in the same route with a time interval. By adjusting several printing conditions (ink concentration, printing parameters, printing time intervals between the two inks, etc.), various structures of composite electrodes, such as layered or fused 2D or 3D structures were developed on the glass substrate. Furthermore, by changing the printing order of the two inks and 3D printer bed temperature, the composite electrodes with a higher printing resolution were successfully printed on the flexible polyimide substrate. The printed composite electrodes via CIW process exhibit the lowest surface electrical resistance of 0.9 kΩ and high flexibility, and stable resistance values were maintained after 1000 cycles of the folding test. Consequently, the CIW process developed in this study applies to the production of the electrical parts and components for various flexible devices, such as foldable and wearable electronics.

## 1. Introduction

Currently, various developments for flexible electronic devices are in progress while the fourth industrial revolution, centered on the Internet of Things (IoT), is underway. Recently, Samsung Electronics has been releasing fully foldable smartphones. Therefore, for most electronic devices of the future, the flexibility of the plastic substrate and the robust interfacial bonding between the substrate and the conductive parts have become essential requirements. Furthermore, to operate the conductive parts in such an environment, other material requirements, such as high conductivity, flexibility, durability, environmental stability, etc. should be simultaneously satisfied [1,2].

In particular, electrode materials used in foldable devices should have excellent flexibility and reliability to maintain electrical properties even under repeated deformation. In the past, a metal thin film or printed metal wiring was utilized, and a metal oxide such as indium tin oxide (ITO) was mainly used as a transparent electrode. However, owing to their low yield strain and unique brittle characteristics, both metal-based materials and ITO show micro-cracks, resulting in a gradual increase of electrical resistance during repeated rapid deformation such as folding [3]. Recently, research on silver nanowires that show high transmittance and electrical conductivity has been actively conducted [4]. However, silver nanowires have disadvantages in that the degree of recovery after repeated large deformation is weak and adhesion to flexible printed circuit board (FPCB) substrates such as polyimide is low. Therefore, the development of new flexible electrode materials and a deposition method onto a plastic substrate are urgently required.

Conducting polymers have high electrical conductivity and, unlike metals, can be wet-processed, so they have the potential to be used in various fields such as flexible electronics [5], bio-electronic materials [6], and energy storage devices [7]. In particular, commercially available poly(3,4-ethylenedioxythiophene):poly(styrenesulfonate) (PEDOT:PSS) is one of the most commonly used conducting polymers, which shows optical transparency at visible range, tunable electrical conductivity, and high flexibility, by incorporating highly conducting PEDOT with positive charges and water-soluble PSS with negative charges [8]. Although PEDOT:PSS aqueous solutions have been widely employed to prepare hole transport/injection layers in lab-size electronics [9], it is necessary to solve the problems of low conductivity compared to metal and oxidation in air, to be applied as a flexible electrode material for industrial electronic applications [10].

Post-modification and preparation of composites are two of the most feasible approaches to effectively improve the electrical properties of PEDOT:PSS polymer. Electrical conductivity (σ) of the bare PEDOT:PSS films can be increased by two or three orders of magnitude through the incorporation of polar solvents [11] and strong acids [12]. Another strategy is to combine PEDOT:PSS with high-conductive additives such as metal/metal oxide nanoparticles and carbon materials, such as carbon nanotubes and graphenes [13]. Since graphenes can control solution processibility, particle size, and even electrical conductivities, depending on the synthetic processes [14,15,16,17,18,19,20], they can be applied to flexible electronics-related applications by using various wet-processing methods, such as drop-casting [21], spin-coating [22], spray-coating [23], and inkjet printing [24], together with a PEDOT:PSS solution.

Recently, Seekaew et al. reported the application of ink-jet printing for the preparation of a flexible graphene-PEDOT:PSS gas sensor [25]. In addition, Wu et al. fabricated a double-layered graphene/PEDOT:PSS conductive film and optimized its optical and electrical properties for large-area flexible organic light-emitting diodes [26]. Very recently, Yuk et al. reported the development of the 3-dimensional (3D) printing of the PEDOT:PSS conducting polymer with high resolution and ready integrity with other materials via the direct ink writing (DIW) process of the PEDOT PSS solution [27]. They also demonstrated 3D printing-based fabrication of the high-density flexible electronic circuit. Although various methods have been proposed for forming flexible electrodes using conducting PEDOT:PSS polymer and its composites, they have limitations such as complicated manufacturing processes and high cost.

In this study, a new process of ‘consecutive ink writing (CIW)’ was developed by modifying the conventional 3D printing processes to print conducting polymer composite materials on flexible plastic substrates. Traditionally, in order to apply the composite materials to the 3D printing process, a filament with a very uniform diameter (~1.75 mm) should be manufactured first through an iterative extrusion processes with matrix polymer and carbon-fillers [28]. These processes not only require lots of time, equipment, and man-power, but also have the disadvantage where it is difficult to increase the content of carbon fillers in the polymer matrix by more than 25% on the lab scale. Therefore, we devised a 3D printer that can continuously print PEDOT PSS and graphene inks by modifying the 3D printing process of the DIW method. This new CIW process enables very fast 2D and 3D printing of composite materials with a high content of graphene filler onto various substrates.

According to the various ink formulations and printing conditions, the printing uniformity and carbon dispersity in the PEDOT:PSS matrix were analyzed through scanning electron microscope (SEM) observations. By varying the consecutive printing speed and drying conditions, microstructures and electrical characteristics of the 3D-printed composite electrodes were controlled. In addition, it was confirmed that the 3D printing of PEDOT:PSS and graphene ink is possible not only on rigid glass substrates but also on flexible polyimide substrates via the CIW process. The CIW process developed in this study is expected to be applicable to produce the electrical parts and components for various flexible devices, such as foldable and wearable electronics.

## 2. Materials and Methods

### 2.1. Materials

PEDOT:PSS (conductive grade, 1.3 wt.% dispersion in H_2_O) was purchased from Sigma-Aldrich (Milwaukee, WI, USA). Electrochemically exfoliated graphene (EEG) was prepared using a previously reported method [29]. A 3D printer (Ghost 5, Flying Bear) was modified for the 3D ink printing, and a sterile needle (Kovax-needle, Dongyang Machinery Co., Ltd. Siheung, Republic of Korea) was installed after removing the conventional fused deposition modeling (FDM) nozzle of the equipment. The needles were flattened at the tip using sandpaper. A syringe Pump (NE-1000, NewEra, Kowloon, Hong Kong), syringe (Henke-ject 3mL, Henke Sass Wolf, Tuttlingen, Germany), and a Teflon tube were used to supply the ink to the needle. The contact between the Teflon tube and the syringe was connected with an air one-touch fitting (GPUC 400, ivory, Kowloon, Hong Kong) to prevent ink leakage. All printing processes were performed on a slide glass (Microscope Slides 76 × 26 mm, SciLab, Rungis, France) and a polyimide polymer film (polyimide Kapton film, 25 mm thickness, In Science, Blacksburg, VA, USA).

### 2.2. Direct Ink Writing with PEDOT:PSS Ink

PEDOT:PSS aqueous dispersion (1.3 wt.%) was heated to 90 °C on a hot plate to prepare the PEDOT:PSS inks with various concentrations (from 3.0 to 7.0 wt.%). To maintain the uniform evaporation of the solvent and the dispersity of PEDOT PSS, it was stirred at a constant speed using a stirring bar during the bath heating process. After approximately 30 min of heating and stirring, an optimal concentration of 5.0 wt.% of PEDOT:PSS solution was prepared.

**Figure 1:**
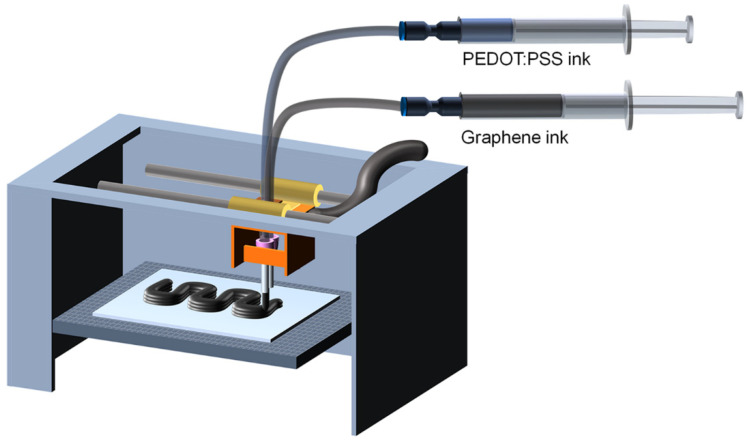
Schematic illustration of the consecutive ink writing with PEDOT:PSS and graphene inks.

The prepared PEDOT PSS ink was cooled down to room temperature and loaded in a syringe for direct ink writing. As shown in Figure 1, an 18G needle (outer diameter of 1.27 mm) was installed on the head of the 3D printer, and the syringe and needle were connected using a Teflon tube. The 3D printer head equipped with the needle moved along the designated G-code path, and at the same time, the syringe pump applied pressure to the syringe at a constant speed to deliver PEDOT:PSS ink along the Teflon tube to the needle. The pumping speed was experimentally tuned (6 mL/min.) according to the concentration of the PEDOT:PPS ink and the moving speed of the 3D printer head, which could print a continuous pattern of the ink on the substrate. During printing, the printing speed was set to 100 mm/s.

### 2.3. Consecutive Ink Writing with PEDOT:PSS and Graphene Inks

EEG aqueous dispersion (1.0 wt.%) was prepared by bath sonication of EEG powder in deionized water for 2 h. The EEG ink with various concentrations was prepared in the same way as the PEDOT:PSS ink. In order to consecutively print PEDOT:PSS ink and graphene ink, two needles were installed in the 3D printer head as shown in Figure 1, and the two inks were printed in the same route with a time delay. The miscibility of PEDOT:PSS and EEG was controlled by adjusting several printing conditions, such as the printing time difference between the two inks, the printing order, and the printer bed temperature. During printing, the pumping speed and printing speed were set as 6 mL/min and 100 mm/s, respectively.

### 2.4. Folding Test of 3D-Printed EEG/PEDOT:PSS Composite Electrodes on Flexible Substrates

Considering the affinity with the flexible polyimide substrate, the EEG ink was first printed, and then PEDOT:PSS was printed in the same route. The two inks were dried in the printer bed for 1 h and then left at room temperature for more than 24 h. Then, 3D printed EEG/PEDOT:PSS composite electrodes on the polyimide substrate were folded several times, and the changes in surface morphology and electrical resistances were measured.

### 2.5. Characterization

The surface morphology of the composite electrodes was investigated using the SEM (CX-200TA, COXEM, Daejeon, Republic of Korea). The detailed surface and cross-sectional SEM images were taken using a field-emission scanning electron microscope (FE-SEM; Mira 3 LMU FEG, Tescan, Brno, Czechia). The electrical resistance of the composite electrodes was measured using the two-probe method (measuring distance of 10.0 mm) by a resistivity meter (FPP-40k, DASOLENG, Cheongju, Republic of Korea). The electrical resistance values from 8 different points were collected and averaged.

## 3. Results and Discussion

### 3.1. CIW of PEDOT:PSS/EEG Inks on Glass Substrates

**Figure 2:**
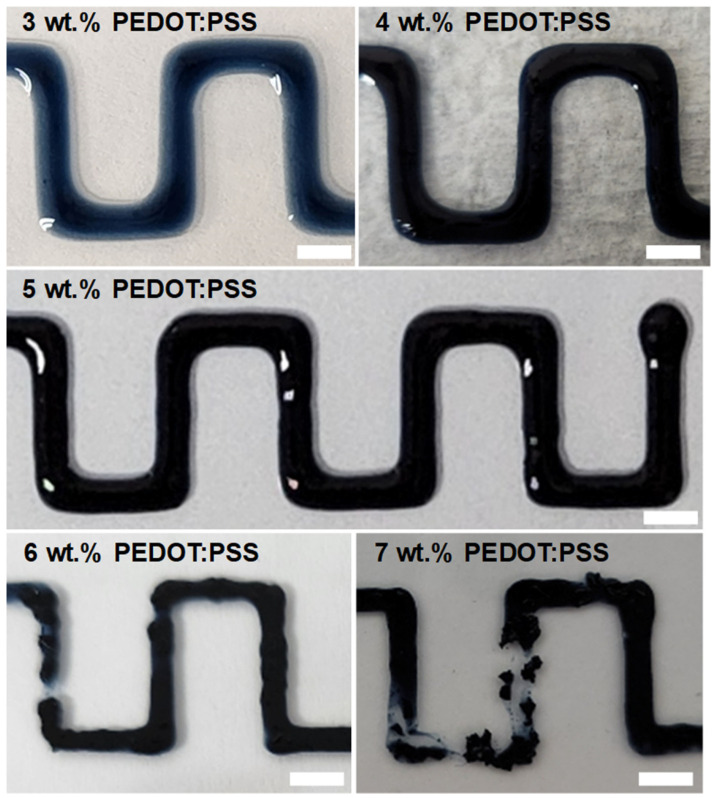
Photo images of the direct ink written-PEDOT:PSS electrodes on the glass substrate according to the PEDOT:PSS ink concentrations. Scale bars: 5 mm.

The surface image of the PEDOT:PSS electrode printed by the DIW process on the glass substrate was observed according to the concentration of the PEDOT:PSS inks (from 3.0 to 7.0 wt.%) (Figure 2). When the concentration of PEDOT:PSS ink was low, such as 3.0 wt.%, the ink spread on the glass substrate due to the low viscosity of the ink and a high affinity between the aqueous solution and the glass substrate. As the concentration of PEDOT:PSS inks increased from 3.0 to 5.0 wt.%, the boundaries of the printed electrodes became clear, and continuous printing was possible with a relatively uniform width. However, when the concentration of PEDOT:PSS inks were more than 6 wt.%, the printed PEDOT:PSS electrodes showed non-uniform thickness and disconnection. Yuk et al. reported that the low viscosity and low yield stress of the PEDOT:PSS inks with low concentrations (1–4 wt.%) caused lateral spreading of 3D-printed inks on the substrate [27]. They also explained that with increasing concentration of the PEDOT:PSS inks, the suspensions gradually transit from liquids to thixotropic 3D printable inks due to the formation of reversible physical networks of the PEDOT:PSS nanofibrils via entanglements within the solvent. Combining our DIW results with the rheological data from Yuk’s research group, the intermediate range of the PEDOT:PSS ink concentrations of 5 wt.% provides optimal rheological properties and DIW printability.

To evaluate the effect of the hydrophilicity of the glass substrate on the printing quality of the PEDOT:PSS electrodes, PEDOT:PSS ink was printed on plasma-treated glass slides. The disconnection of the printed electrodes did not occur even when the concentration of the PEDOT:PSS ink increased to 7.0 wt.%, owing to the generation of hydrophilic functional groups (hydroxyl, carboxylic acids, etc.) on the glass substrate by plasma treatment (Figure S1). However, the resolution of the printing electrodes was severely degraded owing to the excessive spreading of the PEDOT:PSS ink on the plasma-treated glass substrates. It has been reported that a greater surface free energy of solid substrates than that of a liquid induces the wetting and spreading of the liquid on the substrate [30]. Owing the increased surface free energy of the glass substrate and the interaction between the hydrophilic functional groups of the glass and the sulfonic acids of the PSS chains, the PEDOT:PSS ink was excessively wetted onto the glass substrate. Therefore, the following experiments were carried out using untreated glass substrates (just washed with deionized water and acetone) and PEDOT:PSS ink with an intermediate concentration of 5.0 wt.%.

**Figure 3:**
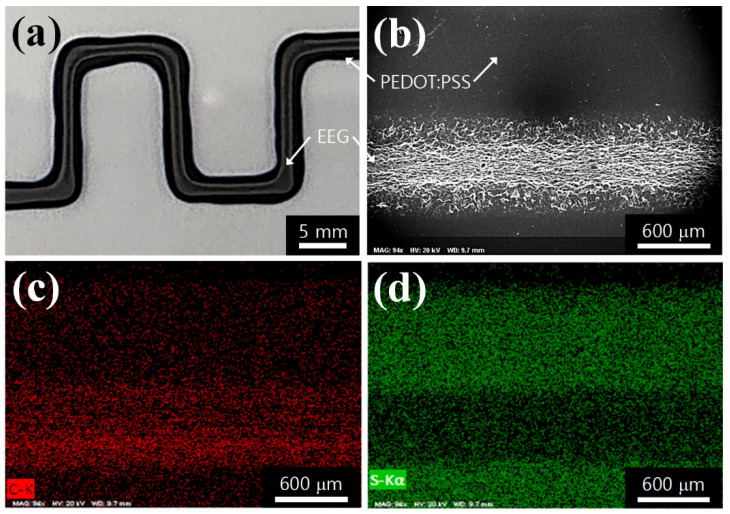
(**a**) Photo and (**b**) SEM images of the consecutive ink written-PEDOT:PSS/EEG electrodes on the glass substrate. (**c**) Carbon and (**d**) sulfur EDS mapping images.

Through the CIW process using the dual nozzles in the 3D printer, the PEDOT:PSS ink and graphene ink were printed in the same path at intervals of printing time. First, the optimized concentration of PEDOT:PSS ink (5.0 wt.%) was printed on the glass substrate by the DIW process, and then dried in a printer bed at room temperature for over 30 min. After that, 3.0 wt.% of EEG ink was printed in the same way and dried for over 30 min. Figure 3a shows that the EEG ink was separately printed on the surface of the PEDOT:PSS electrode. The SEM image also shows that the graphene aggregates were printed on the flat surface of the PEDOT electrode (Figure 3b). Energy-dispersive spectroscopy (EDS) mapping images reveal that the carbon-dominant part forms a thick line of about 600 mm width at the bottom of the image (graphene region), and the remaining part is uniformly distributed with sulfur from the PEDOT:PSS ink (Figure 3c,d). From these observations, it could be confirmed that the PEDOT:PSS and EEG inks did not mix and formed a layered structure.

**Figure 4:**
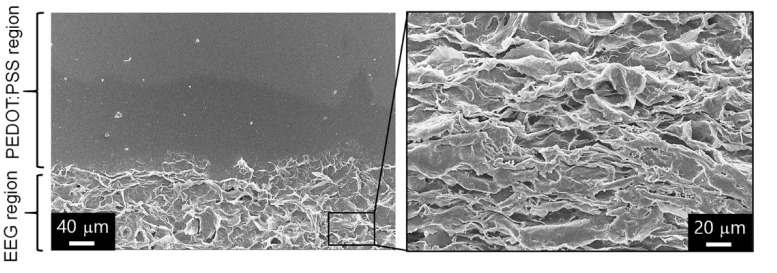
Magnified SEM images of the area where the PEDOT:PSS electrode and the EEG electrode were in contact.

The morphology of the EEG electrode was further analyzed by magnifying the area where the PEDOT electrode and the EEG electrode were in contact (Figure 4). It can be seen that the EEG was rarely dispersed in the background part on which the PEDOT electrode was printed, and EEG was excessively aggregated in the EEG electrode part. A more magnified SEM image shows that the EEG sheets are stacked densely with local alignments along the direction of printing. Our research group has already found that the graphene-only fibers, prepared via wet-spinning of graphene inks, form a stacked structure with wrinkles oriented along the fiber axis direction in a previous study [31]. The stacked structures of graphene sheets could be applied to various fields such as wearable electronic devices and sensors due to their high conductivity and flexibility. However, it is difficult to manufacture graphene-based electrodes with uniform electrical conductivity and high mechanical properties without a polymer binder, owing to the non-uniformity of graphene aggregation. Therefore, as a next step, we tried to manufacture the composite electrodes in which PEDOT:PSS polymer and EEG sheets are fused by tuning the printing conditions (printing time intervals and printing bed temperature, etc.).

**Figure 5:**
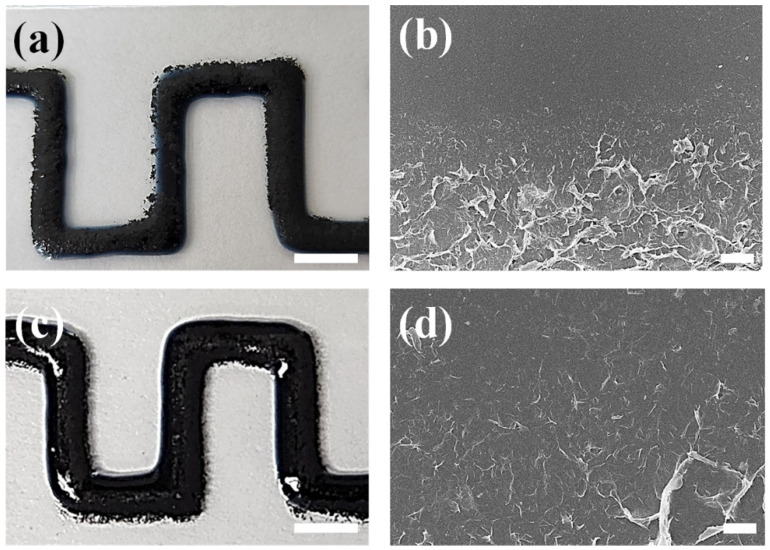
Photo and SEM images of consecutive ink written-PEDOT:PSS/EEG electrodes on the glass substrate. The time interval of 5 min (**a**,**b**), and several seconds (**c,d**). Scale bars: 5 mm (**a**,**c**) and 50 μm (**b**,**d**).

Figure 5 shows the images of the CIW composites according to the printing time interval of the PEDOT:PSS and EEG inks. First, when printing two inks with an interval of 5 min, the PEDOT:PSS and EEG were complexed in some areas. However, excessive agglomeration of EEG sheets was observed in the outer part of the printing line, which happens because the EEG sheets moved to outer part, where the PEDOT:PSS ink was not completely dried (Figure 5a). From the SEM image, the phase separation between PEDOT:PSS and EEG was still clearly observed (Figure 5b).

On the other hand, when the EEG ink was printed immediately after the PEDOT:PSS ink was printed (time interval of several seconds), EEG aggregation or phase separation was not observed (Figure 5c). The SEM image also shows that the EEG sheets are uniformly embedded in the PEDOT:PSS polymer phase (Figure 5d). This result can be explained in two ways: (i) Since both inks were prepared from the same solvent (deionized water) with low concentrations (5.0 wt.% for PEDOT:PSS and 3.0 wt.% for EEG inks), they could be mixed naturally during the printing process. (ii) Since the water soluble PSS part is a good dispersant, it could improve the dispersion of EEG sheets in the PEDOT:PSS polymer matrix [32,33]. However, compared to the previous layered printing structure, we found a problem in which the width of the printing line increased, owing to the in-situ mixing of the two inks during the CIW process. This was solved by changing the substrate and printing order, which will be described in the next part.

**Figure 6:**
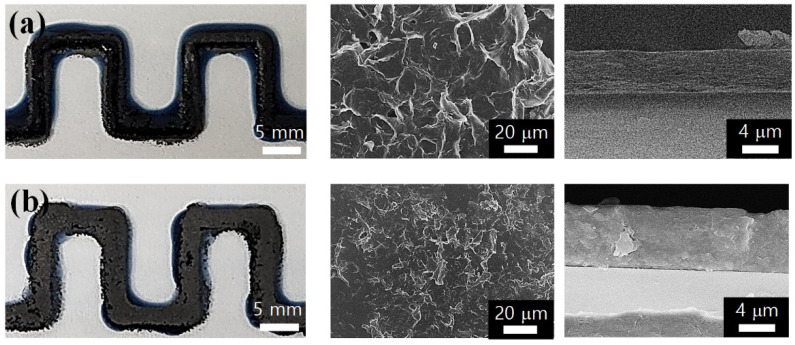
Photo and SEM images (surface and cross-section) of (**a**) P-G-P and (**b**) P-G-P-G composite electrodes on the glass substrate.

In order to apply the CIW process to the 3D printing of composite electrodes, a multi-layer structure with more than two layers was developed using the PEDOT:PSS and EEG inks. Figure 6a shows an printing image of PEDOT:PSS ink once more on the PEDOT:PSS/EEG composite electrode, which will be abbreviated as P-G-P. It can be seen that the printing line path does not deviate, owing to the well-controlled printing conditions (ink concentration, printing speed, time interval between each printing of inks, etc.), even though the number of layers increased. By repeating the same CIW process, 4-layer composite electrodes (P-G-P-G) were successfully printed on the glass substrate (Figure 6b). From the SEM images of the composite electrodes’ surface, it can be seen that the EEG sheets are evenly spread over the PEDOT:PSS matrix of the multi-layer composite electrodes. In addition, the cross-sectional SEM images exhibited that the height of the composite electrodes increased along the *z*-axis through repeated CIW printing (~5.13 μm for P-G-P and ~6.85 μm for P-G-P-G). It should be noted that even though three or four ink printing cycles were repeated in the same route, one fused composite layer was formed without a layer separation, owing to the short time intervals between each printing (several seconds). From these results, it can be concluded that not only 2D but also 3D printing of the composite electrodes is possible through the CIW process.

Table 1 lists the electrical resistance data (measuring distance of 10.0 mm) of the various composite electrodes according to the CIW process conditions. Figure S2 also shows the photo images of the representative electrical resistance values measured from each sample. For the PEDOT:PSS/EEG composite electrode prepared by printing EEG ink after complete drying of PEDOT:PSS ink (briefly abbreviated as PEDOT:PSS/EEG after complete drying), the electrical resistance of each phase was measured due to the phase separation of the two layers. The average electrical resistances of the PEDOT:PSS-rich region and EEG-rich region were measured as 59.7 kΩ and 49.8 Ω, respectively. On the other hand, the PEDOT:PSS/EEG composite electrodes prepared by printing EEG ink before the drying of PEDOT:PSS ink (briefly abbreviated as PEDOT:PSS/EEG before drying) specimen recorded an electrical resistance of 4.5 kΩ. Considering the concentration of the two inks (5.0 wt.% for PEDOT:PSS ink and 3.0 wt.% for EEG ink) and the density of two materials (1.06 g cm^−3^ for PEDOT:PSS and 0.76 g cm^−3^ for EEG), the theoretical electrical resistance value of the PEDOT:PSS/EEG composite is calculated to be approximately 32.6 kΩ, according to the following rule of mixture formula,

*E*_c_ = *E*_m_Φ_m_ + *E*_p_Φ_p_

where, *E*_c_, *E*_m_, and *E*_p_ are the material property (electrical resistance in this case) of the composite, matrix, and particle, respectively. Φ_m_ and Φ_p_ are volume fractions of the matrix and particle, respectively. The lower resistance of the ‘PEDOT:PSS/EEG electrodes prepared before drying’ is thought to be caused by more EEG sheets being distributed on the surface of the composite electrodes, according to the printing order. 

The electrical resistances of the 3D printed-composite electrodes were also measured and are listed in Table 1. The P-G-P composite electrode recorded a slightly higher electrical resistance of 5.63 kΩ compared to that of the PEDOT:PSS/EEG electrodes, presumably because the PEDOT:PSS layer, which has a relatively low conductivity, was printed once more on the composite electrodes. Finally, the 3D-printed P-G-P-G composite electrode recorded the lowest electrical resistance (1.23 kΩ) among the CIW-printed composite electrodes. Through these experiments, it was concluded that the electrical properties as well as the 3D structures of the composite electrodes could be controlled by varying the CIW processing conditions.

**Figure 7:**
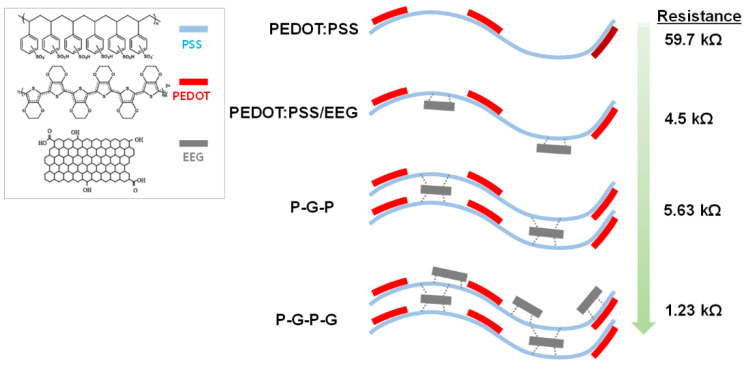
Schematic of the formation of PEDOT:PSS/EEG composite electrode and the multi-layer structures.

Figure 7 displays the schematic of the formation of PEDOT:PSS/EEG composite electrode and the multi-layer structures. The EEG has lower defect density (ratio of the intensity of the D peak to that of the G peak in Raman spectroscopy is 0.14) and much higher C/O ratio (16.2) than those reported for graphene oxide (2.0) and reduced graphene oxide (6.0) [29]. However, small amounts of oxygen-containing functional groups, such as C–OH (4.73 at.%), C=O (2.81 at.%), and C(O)–O (2.43 at.%), were introduced on the graphene sheets during the electrochemical exfoliation of graphite. Due to the interaction between the oxygen-containing functional groups of the EEG and the sulfonic acids of the PSS chains, the PEDOT:PSS/EEG composite electrodes could be formed without the EEG aggregation or phase separation, despite the consecutive printing of the PEDOT:PSS and EEG inks. In addition, this interaction force is thought to act as a bridge in the 3D composite electrodes (P-G-P and P-G-P-G), which could form one fused composite layer, without layer separation, even though three or four ink printing cycles were repeated in the same route.

### 3.2. CIW of PEDOT:PSS/EEG Inks on the Flexible Polyimide Substrate 

To confirm whether the CIW process of the composite inks can be applied to foldable electronics, composite inks were printed on the flexible polyimide substrate. By replacing the substrate from thick and rigid slide glass with thin and flexible polyimide film, the following two advantages were obtained. First, since the very thin polyimide film (25 mm thickness) can transfer the temperature of the 3D printer bed to the printed composite inks directly, it was possible to control the dispersity of the EEG sheets in the composite electrodes according to the bed temperature. Second, through the folding cycle tests, it was possible to measure the changes in the electrical resistance of the composite electrodes, and these results could evaluate the flexibility and long-term durability of the composite electrodes.

To print composite inks on a flexible polyimide substrate through the CIW process, the printing order of the two inks was changed. Owing to the difference in surface-free energies between the as-received polyimide and glass substrates, the spreading of PEDOT:PSS ink occurred on the polyimide substrate (Figure S3) [30]. Therefore, when applying the composite electrodes to the flexible polyimide substrates, the hydrophobic EEG ink was printed first, and then PEDOT:PSS was printed immediately without a time interval (briefly abbreviated as EEG/PEDOT:PSS). This approach reduced the width of the printed line of the composite electrodes (4.5 mm for PEDOT:PSS/EEG to 2.0 mm for EEG/PEDOT:PSS), which was even smaller than the width of the printed composite electrodes on the glass substrate (4.0 mm).

**Figure 8:**
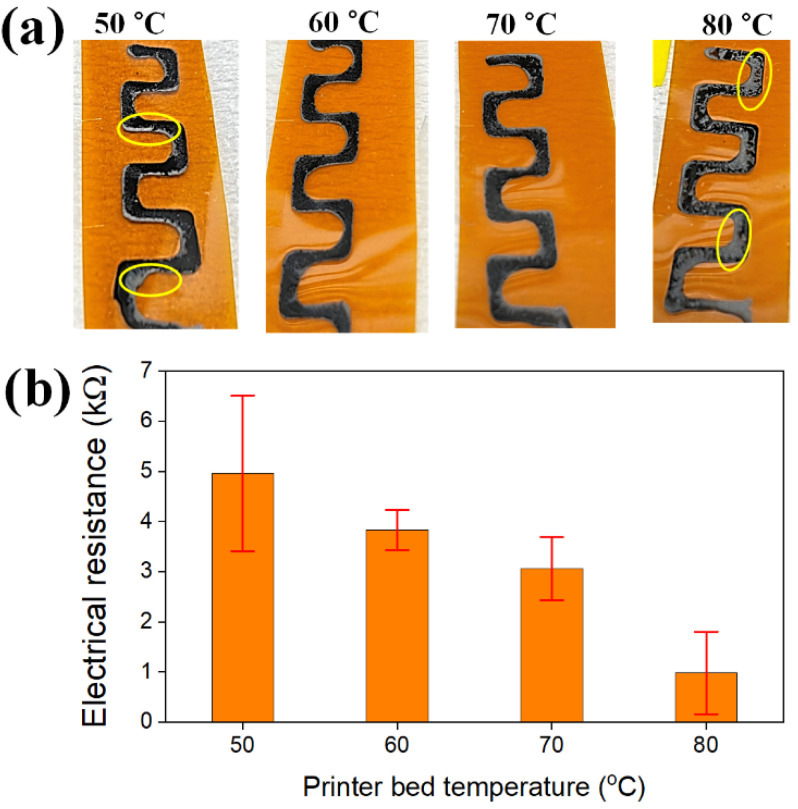
(**a**) Photo images of EEG/PEDOT:PSS composite electrodes on the polyimide substrate according to the bed temperature of the 3D printer. Yellow circles denote the agglomeration of EEG sheets. (**b**) Changes to the electrical resistances and standard deviations of the EEG/PEDOT:PSS composite electrodes.

As a next step, changes in the dispersion state of the EEG sheets in the composite electrodes were investigated according to the bed temperature of the 3D printer. We could print two types of ink at different bed temperatures (room temperature for EEG ink and then controlled temperature for PEDOT:PSS ink) since the bed temperature of the 3D printer rises very quickly and the thin polyimide substrate transfers heat directly to the inks. Figure 8a shows EEG/PEDOT:PSS composite electrodes printed on the polyimide substrate at different bed temperatures (50 to 80 °C). When printed at 50 °C, it can be seen with the naked eye that the EEG sheets are heavily clustered on the edges of the printed line. On the other hand, at the intermediate temperature of 60 and 70 °C, the two inks are uniformly mixed without any EEG agglomeration to form homogeneous composite electrodes. Finally, at the highest bed temperature of 80 °C, EEG ink rose on the surface of the PEDOT ink and formed non-uniform two layers, similar to the PEDOT:PSS/EEG bilayer structure prepared by printing EEG ink after the complete drying of PEDOT:PSS ink.

To quantitatively evaluate the dispersion state of the EEG sheets, the surface electrical resistances of the EEG/PEDTO:PSS composite electrodes were measured. After measuring the electrical resistances at different eight points for each sample, the average values and standard deviation were calculated. As shown in Figure 8b, the composite electrodes printed at the bed temperature of 50 °C recorded both larger resistance (4.96 kΩ) and standard deviation (1.55) than those of other electrodes, because the EEG sheets are insufficient in the measuring points owing to the edge clustering of the EEG. On the other hand, as the printer bed temperature increased, the average resistance value decreased, and finally, the lowest resistance value of 0.98 kΩ was obtained for the composite electrodes prepared at 80 °C. At the high bed temperature, the mobility of the EEG sheets printed on the bottom layer increased, and then immediately rose on the surface of the PEDOT ink by convection (Figure S4), and these results are consistent with the photo image-observations of the composite electrodes at 80 °C. It was noted that, although the resistance values were relatively higher, the standard deviation was the smallest in the composite electrodes printed at 60 °C (0.40). Therefore, from the results of visual observations and electrical resistance measurements, it is concluded that the complexation between the EEG sheets and PEDOT:PSS polymer was best achieved in the printed composite electrodes at the bed temperature of 60 °C.

**Figure 9:**
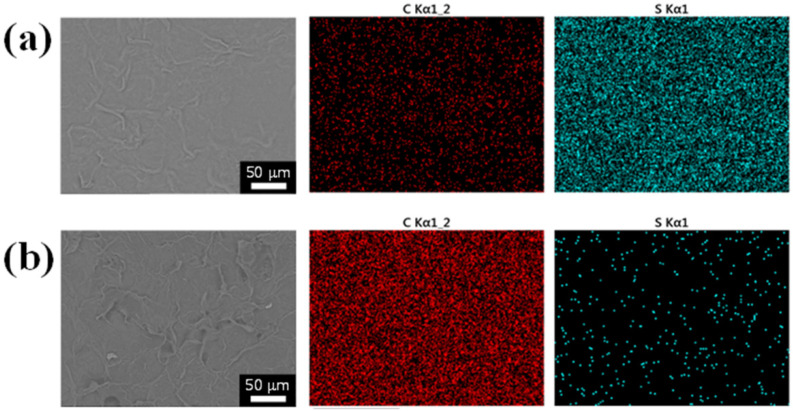
SEM and EDS mapping images of EEG/PEDOT:PSS composite electrodes on the polyimide substrate printed at (**a**) 60 °C and (**b**) 80 °C.

Figure 9 shows the SEM and EDS mapping images of the large areas of the composite electrodes printed at 60 °C and 80 °C. In the composite electrodes printed at 60 °C, it can be seen that the small amount of EEG sheets is well dispersed in the PEDOT:PSS matrix (Figure 9a). In addition, the carbon content detected in the EDS mapping image is much lower than that of sulfur atoms, derived from PEDOT polymers. This is because the EEG ink was first printed on the polyimide substrate and then the PEDOT:PSS ink was printed. On the other hand, the composite electrodes manufactured at 80 °C exhibit a very high carbon content on the surface owing to the circulation of the EEG sheets in the composite inks by heat (Figure 9b). Through SEM observations and the electrical resistance measurements, it was confirmed that the distribution of EEG sheets in the composite electrodes could be tuned by controlling the 3D printing bed temperature.

**Figure 10:**
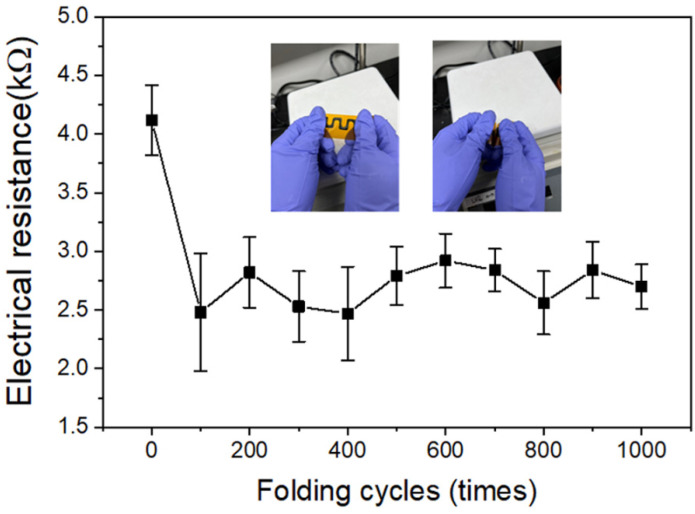
Changes of electrical resistance of EEG/PEDOT:PSS composite electrodes according to the folding cycles. Inset images show the folding test of the composite electrodes on the polyimide substrate.

In order to confirm the applicability of the CIW-printed composite electrodes to the foldable electronic devices, the surface electrical resistances were measured while repeating the folding tests of the EEG/PEDOT:PSS composite electrodes printed on the polyimide substrate. The folding tests were conducted using the EEG/PEDOT:PSS composite electrodes printed at 60 °C, which showed the smooth surface and uniform dispersion of EEG sheets. Figure 10 shows the changes in the surface electrical resistance according to the folding cycles. As a result of several iterative tests, it was found that after the initial 100 folding cycles, the electrical resistance was rather sharply decreased. From the OM image, some comb-shaped wrinkles of the composite material were found at the folded region (Figure S5). It is presumed that the crystallization due to local elongation of the polymer chain or the concentration of the EEG sheets occurred at the folded region. Further research is needed on this. On the other hand, as the number of folding cycles increased by more than 100, a stable resistance value was maintained, and a final value of 2.70 kΩ was recorded after 1000 cycles of folding. Table 2 lists the comparison between the graphene-based inks for the ink writing approaches. Although the direct ink writing of the graphene-based conducting inks has been utilized to develop flexible electronic devices, such as electrochemical energy storage devices [34,35,36,37], to the best of our knowledge, our work is the first trial to develop the foldable electronic devices through the ink writing of the graphene-based ink. Based on these results, it is concluded that the composite electrodes printed on the polymer substrate through the CIW process have enough flexibility and long-term durability to be applied as a part of a foldable electronic device [38]. 

## 4. Conclusions

The PEDOT:PSS and EEG inks were simultaneously printed on both rigid glass and flexible polyimide substrates via the newly developed CIW process for the formation of composite electrodes of foldable electronic devices. When printing the two inks with a time interval of over 30 min, the PEDOT:PSS and EEG inks did not mix and formed a layered structure. On the other hand, when the EEG ink was printed immediately after PEDOT:PSS ink was printed (time interval of several seconds), the composite electrodes were formed without EEG aggregation or phase separation. By repeating this process, the 3D-printed composite electrodes with a height of 6.85 mm and an electrical resistance value of 1.23 kΩ could be manufactured. In addition, the EEG/PEDOT:PSS composite electrodes were successfully printed on the flexible polyimide substrate by changing the printing order of the two inks and the 3D printer bed temperature, which reduced the width of the printed line from 4.0 to 2.0 mm. The printed composite electrodes via the CIW process also exhibited stable resistance values after 1000 cycles of folding tests. Consequently, the CIW process is applicable to the production of composite-based electrical parts for various flexible devices, such as foldable and wearable electronics.

## Figures and Tables

**Table 1:** Average electrical resistances of the PEDOT:PSS/EEG composite electrodes based on CIW conditions.

Sample	Measuring Part	Electrical Resistance (kΩ)
PEDOT:PSS/EEG (after drying)	PEDOT:PSS region	59.7
	EEG region	0.05
PEDOT:PSS/EEG (before drying)	-	4.5
P-G-P	-	5.63
P-G-P-G	-	1.23

**Table 2:** Comparison between graphene-based inks for ink writing approaches.

Graphene	Hybrid Materials	Ink Writing Process	Flexibility Test	Ref.
Graphene oxide	-	Direct ink writing	Bending test (angle ± 60°, 200 cycles)	[34]
Graphene oxide	Polyaniline	Direct ink writing	-	[35]
Graphene oxide	Urea + Gluconic-*δ*-lactone	Direct ink writing	Hand rolling with PET substrate	[36]
Graphene oxide	Ni-Co-O nanosheets	Direct ink writing	-	[37]
Electrochemically exfoliated graphene	PEDOT:PSS	Consecutive ink writing	Folding test (1000 cycles)	This work

## Data Availability

Not applicable.

## Supplementary Materials

The following supporting information can be downloaded at: https://www.mdpi.com/article/10.3390/polym14235294/s1, Figure S1: Photo images of (a) plasma treatment of glass substrate and (b) direct ink written-PEDOT:PSS electrodes on the plasma-treated glass substrate. Figure S2: Photo images of the representative electrical resistance values measured from each sample: (a) PEDOT:PSS-rich region and (b) EEG-rich region of the PEDOT:PSS/EEG after complete drying sample. (c) PEDOT:PSS/EEG before drying, (d) P-G-P, and (e) P-G-P-G composite electrodes. Figure S3: Photo images of (a) PEDOT:PSS/EEG and (b) EEG/PEDOT:PSS composite electrodes printed on the polyimide substrate. Figure S4: Convection of EEG sheets in the printed inks by heating the 3D printer bed. Figure S5: OM image of folded region of EEG/PEDOT:PSS composite electrodes after 100 folding cycles. Yellow dotted circle denotes the comb-shaped wrinkles of the composite material.
