# Supplementary material for: Consecutive Ink Writing of Conducting Polymer and Graphene Composite Electrodes for Foldable Electronics-Related Applications
Source: Polymers (Basel). 2022 Dec 3;14(23):5294. doi: 10.3390/polym14235294 (PMC9736600; doi:10.3390/polym14235294)
Supplement: Supplementary file 1 [file polymers-14-05294-s001.zip › polymers-2063909-supplementary.pdf]

Supplementary data for

Consecutive Ink Writing of Conducting Polymer and Graphene Composite Electrodes for Foldable Electronics-related Applications

Heechan Lee [1,†], Youngdo Kim [2,†], Jiwoo Kim [1], Su Young Moon [3], and Jea Uk Lee [1,*]

[1] Department of Advanced Materials Engineering for Information and Electronics, Integrated Education Institute for Frontier Science & Technology (BK21 Four), Kyung Hee University, 1732 Deogyeong-daero, Giheung-gu, Yongin-si, Gyeonggi-do 17104, Republic of Korea

[2] Samsung Display Co., Ltd, #1 Samsung-ro, Giheung-gu, Yongin-si, Gyeonggi-do 17113, Republic of Korea

[3] Chemical & Process Technology Division C1 Gas & Carbon Convergent Research Center, Korea Research Institute of Chemical Technology (KRICT), 141 Gajeongro, Yuseong, Daejon 34114, Republic of Korea

* Correspondence: leeju@khu.ac.kr (J. U. Lee), Tel.: +82-31-201-3655
† Heechan Lee and Youngdo Kim contributed equally.

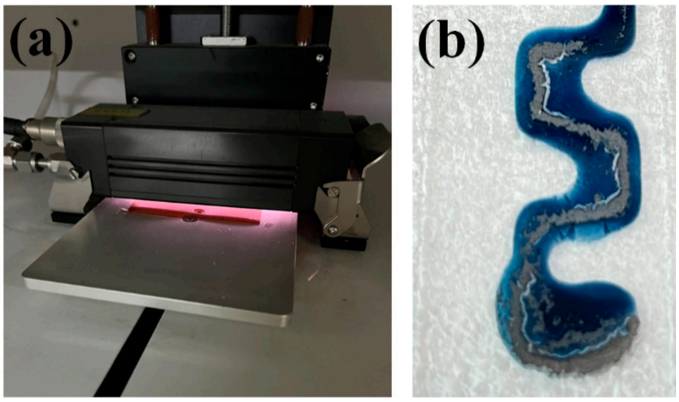

Figure S1. Photo images of (a) plasma treatment of glass substrate and (b) direct ink written-PEDOT:PSS electrodes on the plasma-treated glass substrate.

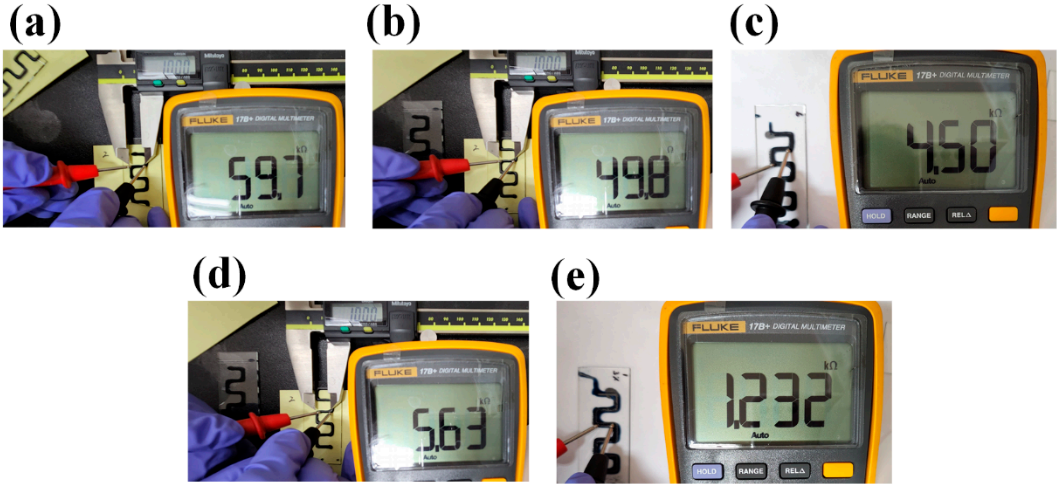

Figure S2. Photo images of the representative electrical resistance values measured from each sample: (a) PEDOT:PSS-rich region and (b) EEG-rich region of the PEDOT:PSS/EEG after complete drying sample. (c) PEDOT:PSS/EEG before drying, (d) P-G-P, and (e) P-G-P-G composite electrodes.

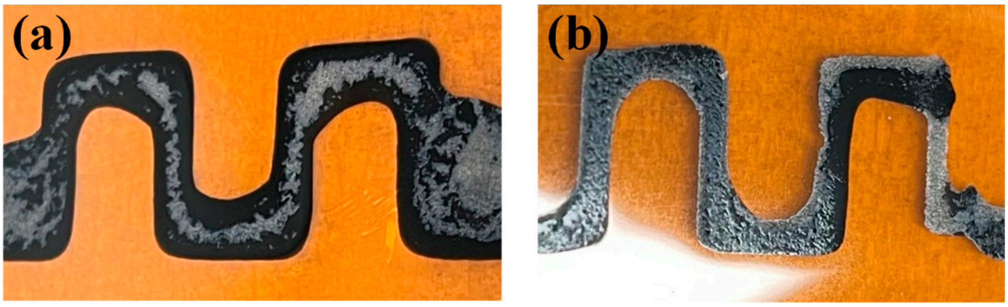

Figure S3. Photo images of (a) PEDOT:PSS/EEG and (b) EEG/PEDOT:PSS composite electrodes printed on the polyimide substrate.

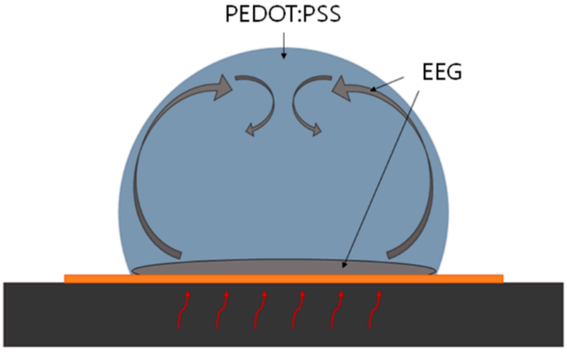

Figure S4. Convection of EEG sheets in the printed inks by heating the 3D printer bed.

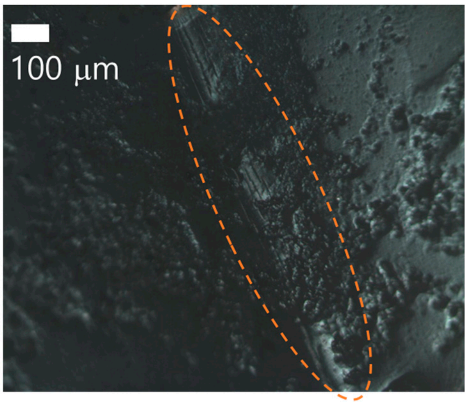

Figure S5. OM image of folded region of EEG/PEDOT:PSS composite electrodes after 100 folding cycles. Yellow dotted circle denotes the comb-shaped wrinkles of the composite material.
